# Optimizing diagnostic and management pathways for patients with eosinophilia of unknown origin: a multidisciplinary protocol for urgent and non-urgent evaluation

**DOI:** 10.3389/fmed.2025.1544047

**Published:** 2025-06-04

**Authors:** Silvia Sánchez-Ramón, Gloria Candelas Rodríguez, Jesús Gimeno Hernández, María Luisa González Gutiérrez, Sissy Fiorella Medina Salazar, Manuel Méndez Bailón, Begoña Navas Elorza, Ángel Nieto Sánchez, Teresa Robledo Echarren, Santiago Rueda Esteban, Marcos Oliver Fragiel Saavedra, María Dolores Zamora Barrios, Cristina Martínez Prada, Celina Benavente Cuesta, Celia Pinedo Sierra, EOS Multidisciplinary Group

**Affiliations:** ^1^Department of Clinical Immunology, Hospital Clínico San Carlos, Madrid, Spain; ^2^Department of Rheumatology, Hospital Clínico San Carlos, Madrid, Spain; ^3^Department of ENT, Hospital Clínico San Carlos, Madrid, Spain; ^4^Department of Allergy, Hospital Clínico San Carlos, Madrid, Spain; ^5^Department of Hematology and Hemotherapy, Hospital Clínico San Carlos, Madrid, Spain; ^6^Department of Internal Medicine, Hospital Clínico San Carlos, Madrid, Spain; ^7^Department of Pediatrics, Hospital Clínico San Carlos, Madrid, Spain; ^8^Department of Internal Medicine (Emergency Room), Hospital Clínico San Carlos, Madrid, Spain; ^9^Department of Pharmacy, Hospital Clínico San Carlos, Madrid, Spain; ^10^Department of Pulmonology, Hospital Clínico San Carlos, Madrid, Spain

**Keywords:** eosinophilia, clinical protocol, patient flow, eosinophilic granulomatosis with polyangiitis, chronic rhinosinusitis with nasal polyposis, hypereosinophilic syndrome, eosinophilic asthma, primary immunodeficiencies

## Abstract

**Introduction:**

Persistent eosinophilia of unknown cause is a key feature of numerous health disorders. These conditions present diagnostic and management challenges, often leading to delayed treatment, increased morbidity and mortality and unnecessary healthcare costs. A systematic approach to patient flow can streamline the process from presentation with eosinophilia to triage management, in hospital settings.

**Methods:**

A proposal of a novel patient flow pathway was developed through a collaborative effort involving 15 diverse multidisciplinary specialists in a public-funded tertiary teaching hospital located in Madrid, Spain, for managing eosinophilic diseases. This pathway focuses on early identification and expedited referral circuits in severe cases of hypereosinophilia and early screening of primary immunodeficient patients to optimize the journey from initial presentation through diagnosis and initial management.

**Results:**

The proposed patient flow model is designed to be replicable in other hospital settings. Its implementation aims to facilitate timely diagnosis and reduce the preventable morbidity, mortality, suffering and economic burden associated with complex eosinophilic conditions.

**Conclusion:**

The development and implementation of a structured patient flow pathway for eosinophilia of unknown cause in a tertiary hospital setting demonstrates a significant step toward improving patient outcomes. This model serves as a template for other healthcare institutions seeking to enhance the management and care of patients with eosinophilic diseases.

## 1 Introduction

Hypereosinophilia characterizes at least five often underdiagnosed conditions, namely: eosinophilic granulomatosis with polyangiitis (EGPA) (1), chronic rhinosinusitis with nasal polyposis (CRSwNP) (2), hypereosinophilic syndrome (HES) (3), severe eosinophilic asthma (SEA) (4) and primary immunodeficiencies (PID) (5).

Prompt diagnosis, coupled with multidisciplinary management is crucial to avert disease progression and potential irreversible organ damage and death (6, 7). However, the rarity (3, 8–12) and diverse symptomatology of these syndromes (1, 13, 14) often lead to late diagnosis (8, 15), after irreversible complications have occurred (14). Patients experiencing these complex conditions, which are commonly poorly understood by generalists (1, 16, 17), experience inefficient referral processes and repeated emergency department (ED) presentations (6).

The high morbidity rates as disease-related complications (e.g., end-organ damage, reduced quality of life) and healthcare burdens (e.g., repeated hospitalizations), are partly due to diagnostic delays and inadequate treatment (6, 17), underscore the importance of early detection (17). Furthermore, literature suggests significant financial implications associated with delayed diagnosis (7, 18). These conditions present diagnostic and management challenges, often leading to delayed treatment, increased morbidity and mortality, and unnecessary healthcare costs.

On the other hand, serum eosinophilia is easily detectable via routine complete blood count (19). Since eosinophilia is a core feature of the conditions discussed above, detection of serum eosinophilia in the ED or at initial hospital presentation represents a unique opportunity to streamline the diagnosis of these underdiagnosed pathologies.

Peripheral eosinophilia, defined as an absolute eosinophil count (AEC) of ≥0.5 × 10^9^/L, can be associated with a variety of conditions. Infections, particularly parasitic infections, are a common cause of eosinophilia, especially in individuals with recent travel history to endemic areas; medications, including nonsteroidal anti-inflammatory drugs (NSAIDs) and antibiotics, are also frequent culprits, often leading to drug-induced eosinophilia and hypersensitivity reactions; hematologic malignancies, such as chronic eosinophilic leukemia (CEL), are another important cause, where eosinophilia may be either reactive or primary, driven by the malignant clone (20). The American Academy of Allergy, Asthma, and Immunology emphasizes the importance of a thorough diagnostic workup to identify the underlying cause and guide appropriate management (21).

The overlap of symptoms between different causes of eosinophilia and the potential for organ damage necessitates a thorough and systematic evaluation, often involving multiple specialties. This multidisciplinary approach involves collaboration across nine different specialties such as pediatrics, allergy, hematology, immunology, rheumatology, ENT, pharmacology, emergency, and internal medicine to ensure comprehensive evaluation and treatment.

Although we do not have our current data, we assume similar diagnostic delays and misdiagnosis rates due to fragmented referrals as those reported in the literature: the median diagnostic delay for both EGPA and HES is approximately 18 months; and a misdiagnosis rate of approximately 23% for EGPA and HES (17, 22). These numbers highlight the urgent need for heightened clinical awareness and an integrated evaluation of patients with eosinophilia and systemic symptoms.

We propose a patient flow pathway to expedite the identification and management of patients with persistent eosinophilia through a multidisciplinary team at a tertiary hospital in Madrid, Spain. This pathway streamlines referrals from initial presentation to diagnosis and initial management of suspected severe cases, ensuring detailed assessment and department-specific interventions. This model, aimed at reducing preventable morbidity, mortality and healthcare costs, is replicable in other healthcare settings.

## 2 Assessment of policy/guidelines options and implications

### 2.1 Methodology

The patient flow presented in this manuscript was constructed in phases and through a process of co-creation between the authors, members of 10 different services involved in the management of eosinophilic diseases at the Hospital Clínico San Carlos, Madrid, Spain [medical specialists in: Allergology (2), Emergency Medicine (1), Hematology (2), Immunology (1), Internal Medicine (2), Otolaryngology (1), Pediatrics (1), Pulmonology (1), and Rheumatology (2) and Hospital Pharmacy (1)].

Step 1: Pre-task (March 2021).

Online questionnaire to collect base information for creating version 0 of the circuit.

Step 2: Working meeting (April–June 2021).

A working group meeting took place to define the next steps, from the starting patient:

a) Screening via urgent triage

The triage categories for patients with eosinophilia align with the current understanding and classification of eosinophilic disorders according to the World Health Organization (WHO) and the International Consensus Classification (23, 24):

- Urgent (<30 days): This category is for patients with hypereosinophilia (≥1.5 × 10^9^/L) and signs of organ damage, such as cardiac or neurological involvement. There is a need for immediate intervention to prevent irreversible organ damage.
- Semi-urgent (30–90 days): This category includes patients with persistent eosinophilia (≥1 × 10^9^/L) without organ involvement. The WHO guidelines suggest a watch-and-wait approach with close follow-up.
- Non-urgent (>90 days): This category is for transient eosinophilia or mild cases with no systemic features. The WHO guidelines support less immediate intervention for these patients, focusing on monitoring and addressing any underlying causes if identified.

b) Differential diagnosis of EGPA, CRSwNP, HES, SEA and PID, including the multidisciplinary team in charge of management, using cited diagnostic criteria (e.g., ACR 2022 for EGPA, IUIS 2020 for PID) and guidelines (e.g., NIH HES Consortium 2019).

Step 3: Post-task (April–June 2021).

One-hour virtual meetings were held to solidify the aspects of the circuit.

Step 4: Co-creation (April–June 2021).

Drafting and review of the patient flow was completed by circuit authors.

Step 5: Manuscript preparation (July–September 2021).

Drafting and review of the manuscript was completed by all authors.

### 2.2 Results

The patient flows generated are presented in a sequential manner.

#### 2.2.1 Target patient

Initially, the target patient with a potential suspicion of disease presenting with eosinophilia should be identified. The target patient is defined as follows: a patient with persistent eosinophilia (≥1 × 10^9^/L), or hypereosinophilia (i.e., ≥1.5 × 10^9^/L) in at least two determinations in an interval of 3–6 months, without any other known cause, or presenting organ damage, or presenting with eosinophilia with a clear clinical cause. The target patient would also be included if they have >500 cells/μL and they have previously received any medication that may have reduced the blood eosinophil count, such as systemic glucocorticoids, biologics targeting IL-5, hydroxyurea or interferon-*α* (25). This target patient might access the hospital via different routes, but regardless of the patient’s origin, the procedure defined in this manuscript should be applied when dealing with a patient with the aforementioned characteristics.

Transient eosinophilia should be excluded by repeating the serum measurement over an interval of 3–6 months, because transient and relatively benign causes such as atopic allergy are common (25).

If, in addition to an absolute eosinophil count of ≥1 × 10^9^/L and particularly if eosinophilia ≥1.5 × 10^9^/L, any of the following features are present, the suspicion of a systemic disease with hypereosinophilia will be strengthened:

- Poorly controlled asthma, not classified as severe eosinophilic asthma (26)
- Persistent dyspnea, irrespective of the eosinophil count at any given time (27)
- Dysphagia
- Neurological symptoms (e.g., polyneuropathy, paresthesia, etc.) (28)
- Target organ involvement (29)
- Urticaria or non-infiltrative skin lesions (suspected vasculitis) with recurrent presentations at the ED within a short period of time, regardless of the eosinophil count at any given time.
- Chronic diarrhea or digestive discomfort
- Arthralgia or myalgia (30)
- Fever (30)

No matter whether the above features are present or not, all target patients should proceed to the initial analytical protocol, as outlined below.

#### 2.2.2 Initial analytical protocol

All target patients will proceed down the following initial analytical protocol, although any emergency situations should first be addressed, and stabilization should be sought.

##### 2.2.2.1 Initial assessment

A full medical history will be taken, and initial investigations ordered. While awaiting test results, the patient will be seen by the service that is monitoring and ordering the investigations.

**Figure 1:**
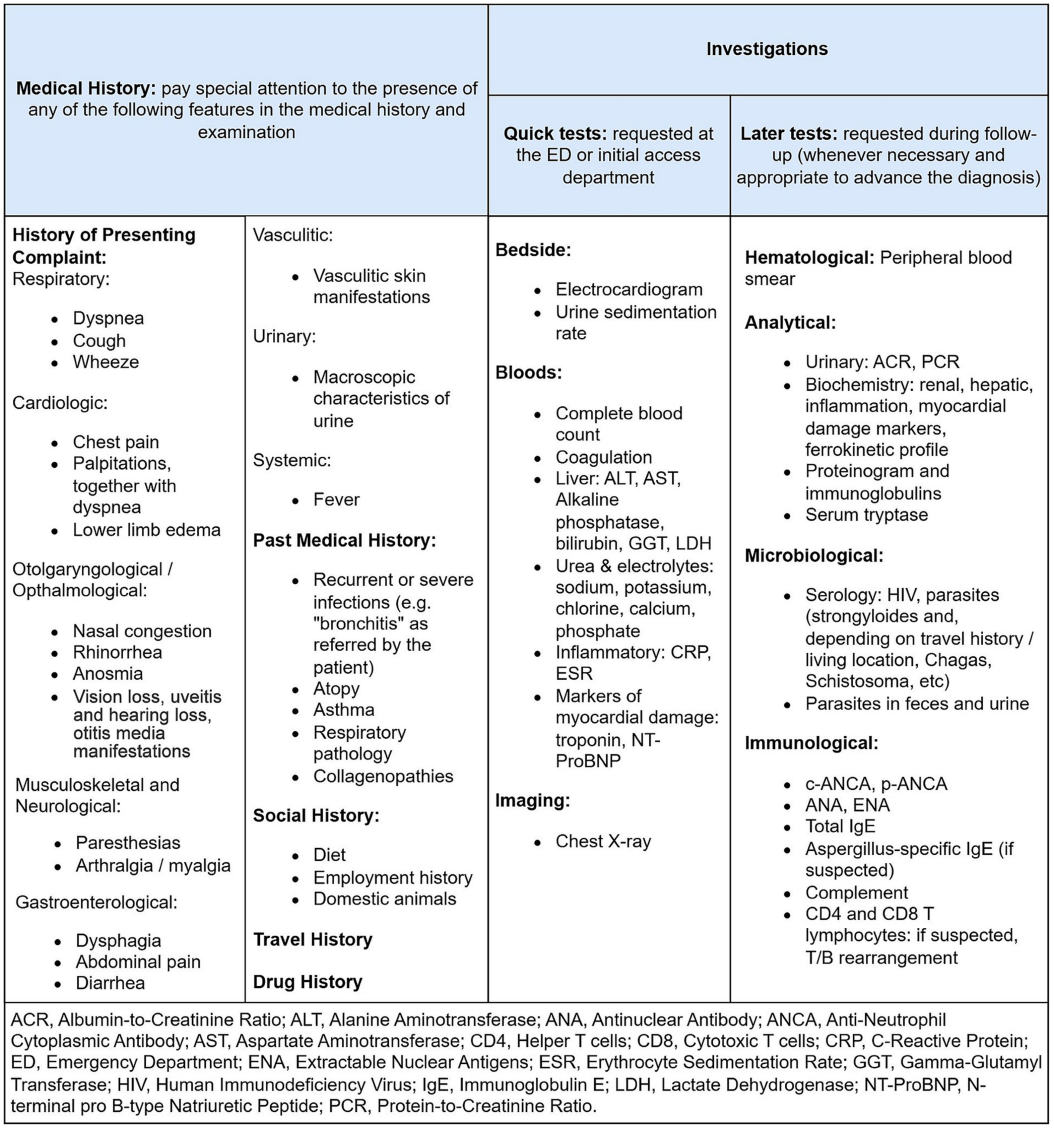
Initial assessment to be performed for the target patient, including medical history findings to be sought and investigations to be ordered.

Figure 1 defines the key features in the medical history and examination that should be actively sought, that may point to the presence of an eosinophilic condition. The figure also defines the investigations that should be carried out, either in the ED, at the patient’s initial access department, or during follow-up.

##### 2.2.2.2 Referral routes and consultations

If the patient presents to the ED, blood tests including a complete blood count, biochemistry and coagulation should be performed in the ED, as well as a chest X-ray and an electrocardiogram. The location where the remaining investigations should take place, as outlined in Figure 1, will depend on whether the patient requires admission or not. If admission is required, the department to which the patient is admitted shall be responsible for completing the remaining investigations; if not, follow-up in outpatient clinic will be scheduled, where these investigations shall be completed.

If the patient is admitted to hospital, they will be referred to the Internal Medicine Service, except if a very specific organ is affected, as outlined below:

- At the time of triage, consultations should be sought with Hematology and/or Clinical Immunology in case the following features are noted:
  ○ The on-call hematologist should be consulted first if the patient presents with hypereosinophilia.
  ○ Clinical Immunology should be consulted if any of the following features are present:
- >2 episodes of sinusitis in 1 year
- >4 otitis in 1 year
- >2 radiologically confirmed pneumonias in 1 year
- Meningitis or severe infection
- Persistent infections or infections requiring intravenous antibiotic treatment
- Autoimmune diseases
- Organomegaly
- Growth or development failure
- Dermatitis
- Syndromic phenotype
- Rheumatology consultation is prioritized for patients with arthralgia, myalgia, or vasculitic rash to assess for EGPA or connective tissue disease.
- Pneumology and/or allergy service if respiratory pathology predominates. Respiratory pathology: Dyspnea, wheezing, or radiographic infiltrates suggestive of eosinophilic pneumonia.
- Allergy service if allergic pathology predominates. Allergic pathology: Recurrent urticaria, anaphylaxis, or IgE-mediated reactions.

While the investigations within the initial analytical protocol are underway (Figure 1), the absolute eosinophil count shall be repeated weekly to confirm that it remains equal or above 1 × 10^9^/L (equal or more than 1,000 cells/μL).

##### 2.2.2.3 Differential diagnosis

**Figure 2:**
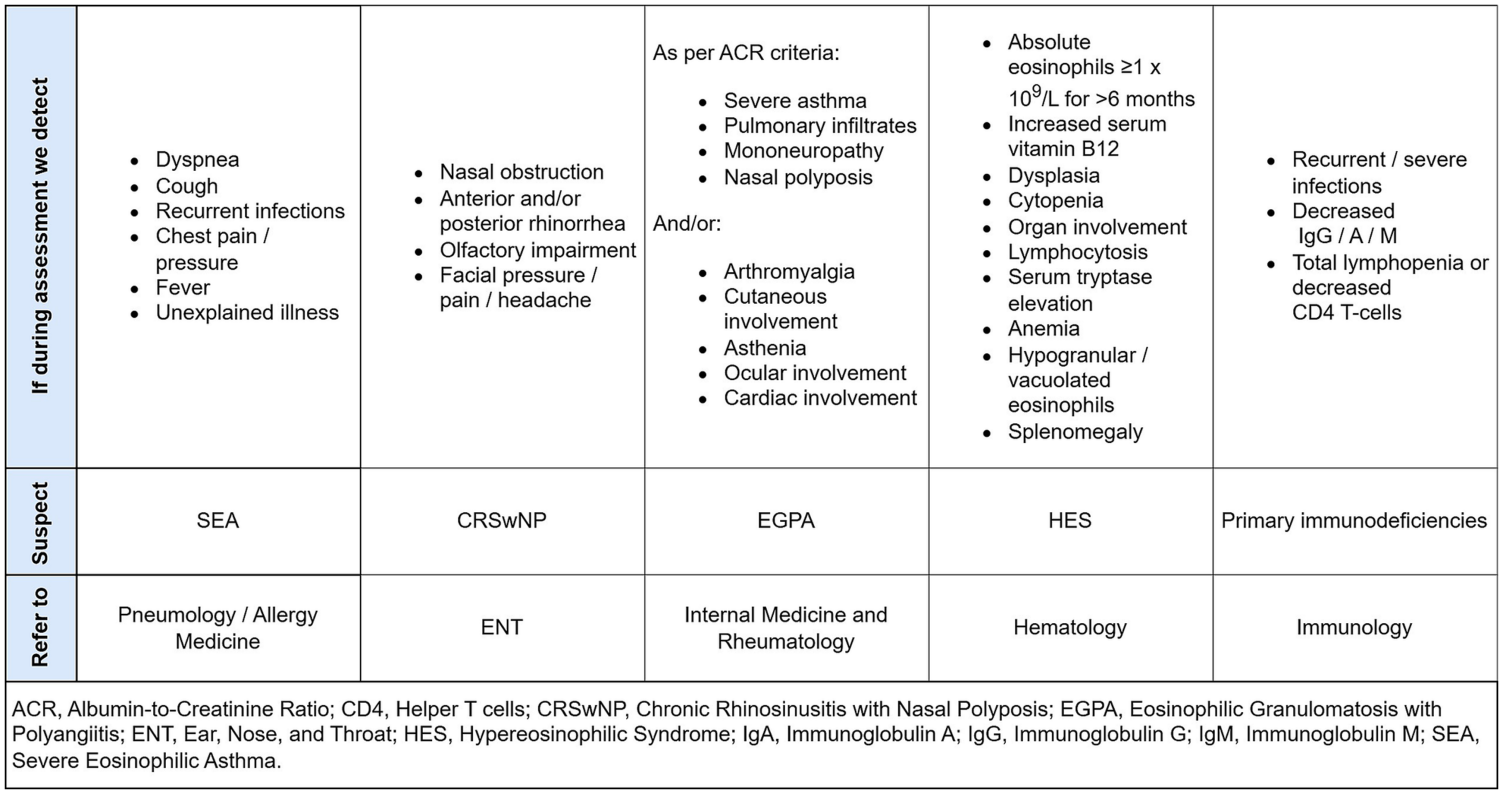
Features detected during assessment that leads us to suspect an eosinophilic differential diagnosis, and which department to refer the patient to confirmed diagnosis.

Figure 2 describes the process by which an initial differential diagnosis is made, and the department to which the target patient should then be referred.

Within each department there should be a figure with specific training in the respective pathologies, who should be responsible for this differential diagnosis process to optimize resources and avoid delays and duplication. If SEA, CRSwNP, EGPA, HES or PID are excluded, the patient should be followed up by the Internal Medicine service or Primary Care.

Next, the management flows that are specific to each condition shall be presented.

#### 2.2.3 Hypereosinophilic syndrome flow

**Figure 3:**
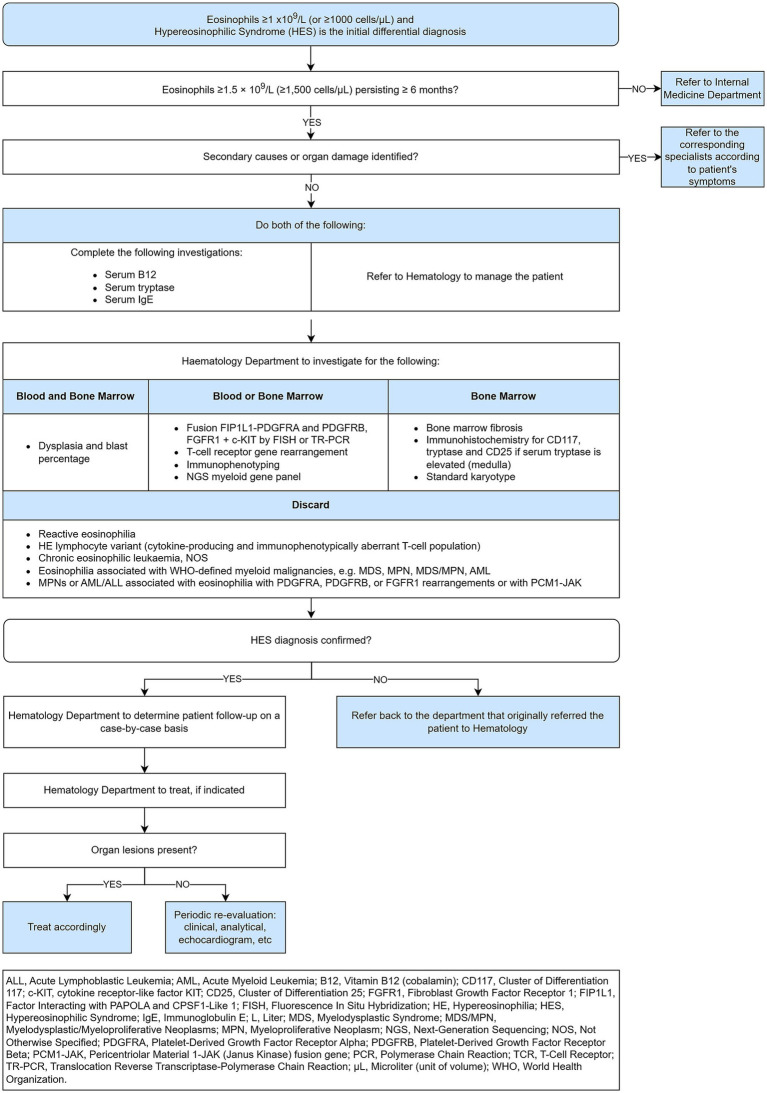
Hypereosinophilic syndrome flow, from initial suspicion to confirmed diagnosis.

HES is a rare disease that is underdiagnosed (29). Establishing a clear starting point for the diagnostic process can significantly aid in its identification and management. In a patient with suspected HES, the process described in Figure 3 should be implemented to transition from initial suspicion to confirmed diagnosis.

HES is characterized by persistent peripheral blood eosinophilia (≥1.5 × 10^9^/L) and evidence of end-organ damage. The clinical presentations of HES are diverse, ranging from asymptomatic eosinophilia to severe, life-threatening conditions such as endomyocardial fibrosis and thromboembolic events. Patients with HES may present with a variety of symptoms, including fatigue, fever, weight loss, and organ-specific manifestations such as cardiac, pulmonary, gastrointestinal, dermatologic, and neurologic involvement. Cardiac involvement, including myocarditis and endomyocardial fibrosis, is particularly concerning due to its association with significant morbidity and mortality (23). The 2022 International Consensus Classification (ICC), as detailed by Wang et al., establishes a structured diagnostic score for HES that integrates clinical, hematologic, and molecular parameters to enhance diagnostic precision and disease classification (23). Central to this approach is the identification of persistent peripheral eosinophilia, defined as an absolute eosinophil count ≥1.5 × 10⁹/L, along with evidence of organ damage directly attributable to eosinophilic infiltration—both of which are considered major diagnostic criteria. The ICC further mandates a comprehensive exclusion of secondary causes, including parasitic infections, allergic conditions, and drug-induced eosinophilia, as a critical step before confirming a diagnosis of HES. Diagnostic refinement is achieved through bone marrow morphological analysis, cytogenetic and molecular profiling (including FISH and NGS), and flow cytometric immunophenotyping to identify clonal eosinophilia or underlying hematologic malignancies such as aberrant T-cell populations. Importantly, the ICC also delineates distinct genetic markers and bone marrow features to differentiate idiopathic HES from chronic eosinophilic leukemia not otherwise specified (CEL, NOS) (23). This multi-tiered scoring system—with major and minor criteria—ensures a thorough diagnostic workup, supporting timely and accurate classification that guides optimal therapeutic strategies for patients with eosinophilic disorders (27). Follow-up should include monitoring of eosinophil counts, assessment of organ function, and evaluation for potential complications. Treatment response and disease progression should be regularly assessed, with adjustments to therapy as needed. First-line treatment for HES typically involves corticosteroids, which are effective in reducing eosinophil counts and mitigating organ damage. For steroid-refractory cases, other options include hydroxyurea, interferon-*α*, and targeted therapies such as mepolizumab (an IL-5 antagonist) and imatinib for PDGFRA/B-rearranged HES (23, 24). The AAAAI emphasizes the importance of cytogenetic and molecular testing in the workup of eosinophilia. Peripheral eosinophilia and HES are associated with various genetic mutations that influence eosinophil proliferation and function. Recent studies have identified several key genes involved in these conditions. Commonly mutated genes in IHES include ASXL1, TET2, EZH2, SETBP1, CBL, NOTCH1, SCRIB, STAG2, SH2B3, PUF60, CDH17, LMLN, AQP12A, and PCSK1. These mutations suggest clonality and may lead to reclassification of IHES cases as CEL, NOS (21).

##### 2.2.3.1 Secondary causes of eosinophilia that should be ruled out

Eosinophilia can be a relevant marker in various clinical settings, including parasitic infections, drug reactions, and specific hematologic malignancies. Eosinophilia is commonly seen in parasitic infections, such as strongyloidiasis and toxocariasis. Strongyloidiasis, caused by Strongyloides stercoralis, often leads to eosinophilia due to the parasite’s tissue-invasive nature, which triggers an immune response (31). Toxocariasis, caused by Toxocara canis or Toxocara cati, also results in eosinophilia, particularly in cases with organ involvement (32).

Certain drugs, most commonly beta-lactam antibiotics and anticonvulsants, are known to induce eosinophilia, which can be severe and even life-threatening (33, 34). Beta-lactams, such as penicillins and cephalosporins, can cause drug-induced eosinophilia and hypersensitivity reactions, including rash and renal failure. Anticonvulsants like valproate and carbamazepine can also lead to eosinophilia, potentially through mechanisms involving interleukin-5 (35). Eosinophilia can be a feature of certain malignancies, particularly myeloproliferative neoplasms associated with platelet-derived growth factor receptor alpha (PDGFRA). These neoplasms, such as chronic eosinophilic leukemia with FIP1L1-PDGFRA fusion, are characterized by clonal eosinophilia and often respond well to tyrosine kinase inhibitors like imatinib (36).

#### 2.2.4 Eosinophilic granulomatosis with polyangiitis flow

EGPA is associated with impaired quality of life and high healthcare resource utilization, including inpatient admissions and ED visits (12, 37).

The 2022 American College of Rheumatology (ACR) and European Alliance of Associations for Rheumatology (EULAR) classification criteria for Eosinophilic Granulomatosis with Polyangiitis (EGPA) include several key clinical features and laboratory findings, including: (i) nasal polyps and asthma are common in EGPA and help differentiate it from other vasculitides. Nasal polyps are assigned a weight of +3 points, reflecting their relevance in the diagnosis. Asthma is also heavily weighted at +3 points. (ii) Antineutrophil Cytoplasmic Antibody (ANCA) Positivity, in particular cytoplasmic ANCA (c-ANCA) or anti-proteinase 3 (PR3-ANCA), is included in the criteria with a weight of −3 points, reflecting the fact that ANCA positivity is less common in EGPA compared to other ANCA-associated vasculitides like granulomatosis with polyangiitis (GPA) and microscopic polyangiitis (MPA) (38). ANCA testing, particularly for myeloperoxidase-ANCA (MPO-ANCA), has a sensitivity of approximately 40% in EGPA patients, and is associated with more vasculitic features such as glomerulonephritis, neuropathy, and skin manifestations (39).

EGPA is a heterogeneous disease and this heterogeneity, and the wide range of clinical manifestations often results in a prolonged delay to diagnosis (40). This makes the constitution of a coordinated working and management group necessary to manage patients with the potential to be diagnosed with EGPA.

**Figure 4:**
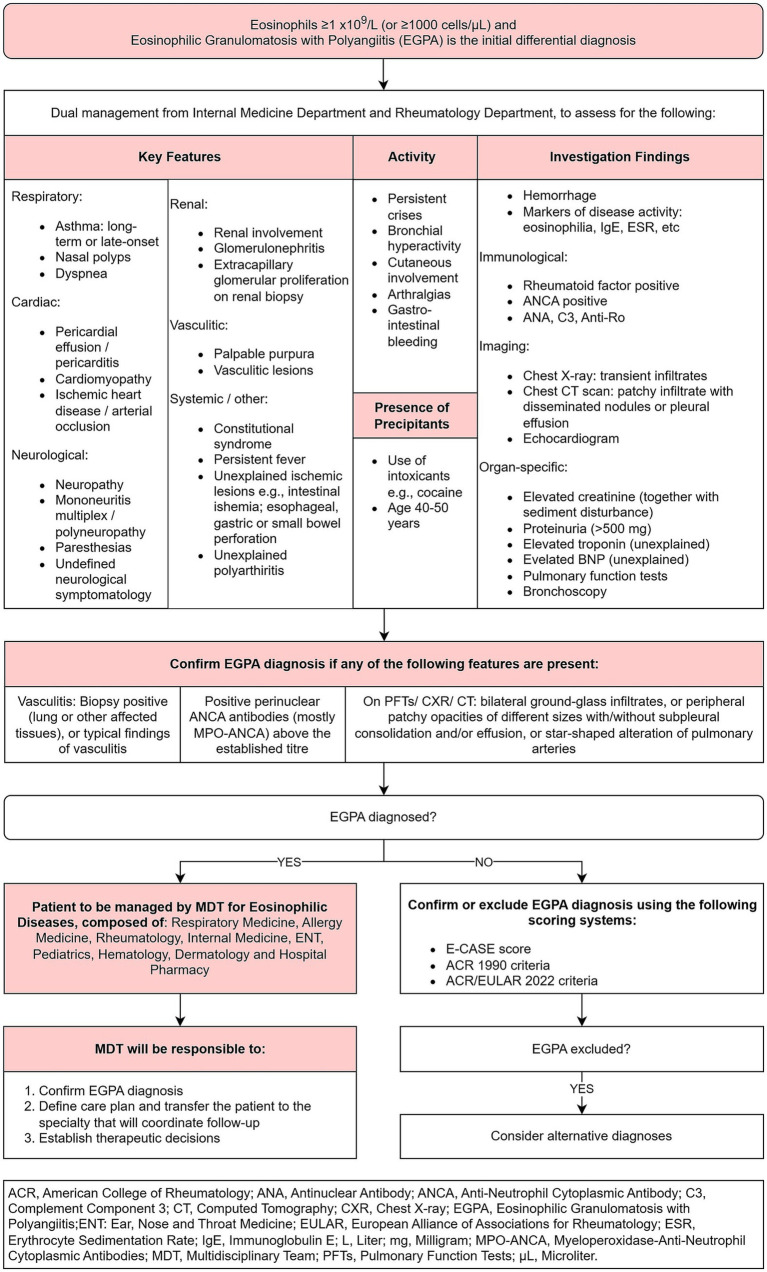
Eosinophilic granulomatosis with polyangiitis flow, from initial suspicion to confirmed diagnosis.

In patients with suspected EGPA, the process illustrated in Figure 4 should be implemented to guide the transition from initial suspicion to confirmed diagnosis.

#### 2.2.5 Primary immunodeficiencies flow

**Figure 5:**
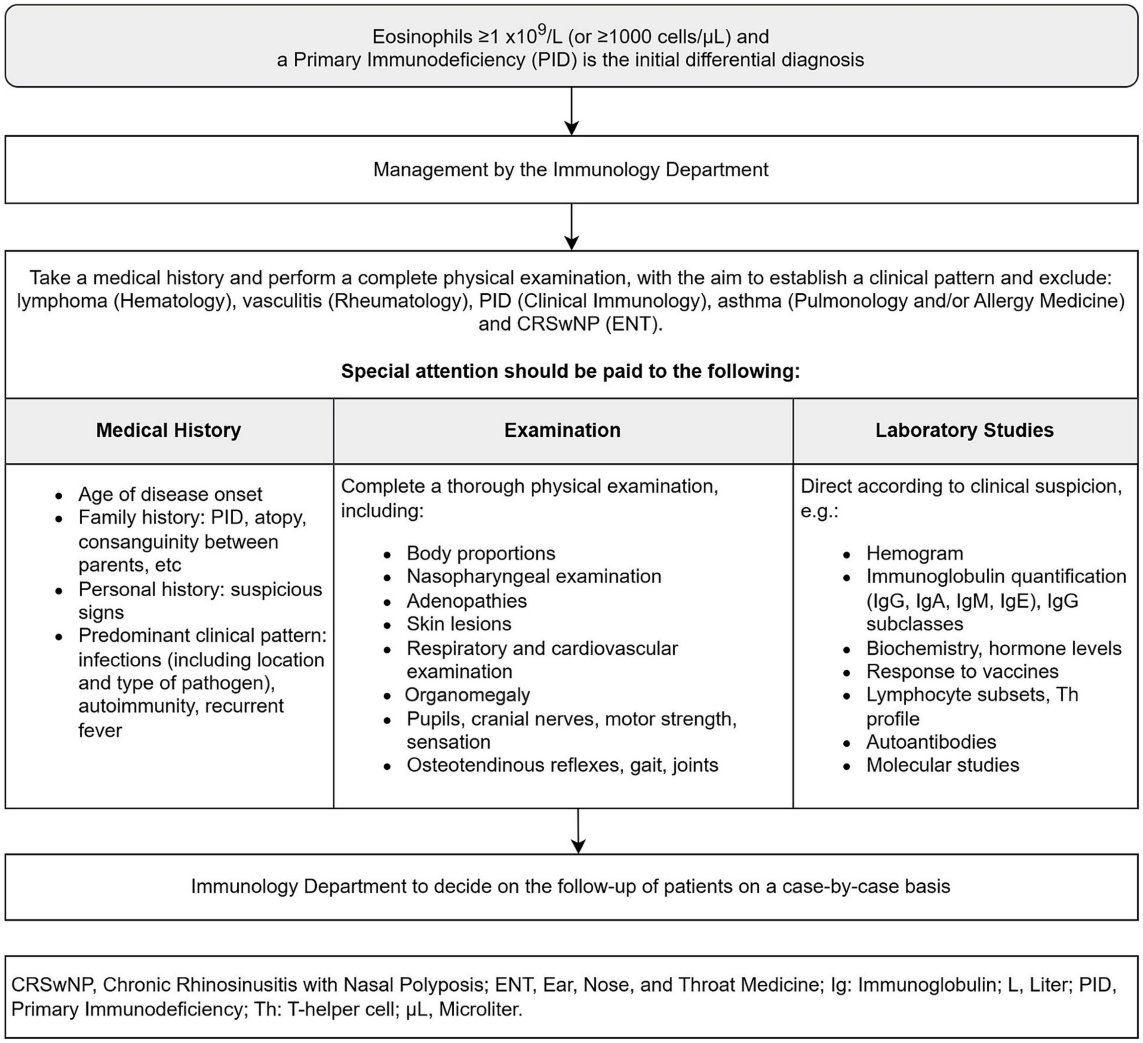
Primary immunodeficiencies flow, from initial suspicion to confirmed diagnosis.

PIDs are a rare and heterogenous group of more than 500 monogenic disorders that present with diverse symptomatology and complications, such as recurrent infections, autoimmune or inflammatory complications, allergies and cancer (41, 42). Indeed, it is estimated that 70–90% of patients with PID remain undiagnosed (42). Although only subgroup of PIDs present with eosinophilia, the literature emphasizes the importance of investigating PIDs in patients who present with eosinophilia, in whom common causes are excluded (5, 43). Therefore, we recommend that a patient with suspected PID should follow the pathway outlined in Figure 5. This includes clinical assessment, laboratory testing, and genetic analysis to identify underlying molecular defects. Accurate differential diagnosis is crucial to avoid misdiagnosis and ensure appropriate treatment.

Eosinophilia is a hallmark in several IEIs, particularly those associated with defects in immune regulation and T-cell function. Autosomal dominant STAT3 loss-of-function mutations, characteristic of STAT3 hyper-IgE syndrome (STAT3-HIES), and autosomal recessive DOCK8 deficiency are prototypical IEIs presenting with elevated eosinophil counts and elevated serum IgE levels (43, 44). In STAT3-HIES, patients exhibit a triad of eczema, recurrent skin and pulmonary infections, and high IgE levels, often accompanied by connective tissue and skeletal abnormalities. Eosinophilia in this syndrome is linked to impaired Th17 cell differentiation, which compromises mucocutaneous immunity. In contrast, DOCK8 deficiency, while also presenting with severe atopy and very high IgE, is characterized by marked T-cell lymphopenia, especially of naïve CD8^+^ T cells, and a pronounced susceptibility to cutaneous viral infections, such as molluscum contagiosum and herpes simplex virus. Eosinophilia in DOCK8 deficiency reflects immune dysregulation and contributes to the severity of allergic manifestations. Additionally, patients often show reduced memory B cells, defective NK cell function, and are at increased risk for malignancy. Other IEIs associated with hypereosinophilia or eosinophilia are phosphoglucomutase 3 (PGM3) deficiency, adenosine deaminase Immunodeficiency, Omenn syndrome, Loeys-Dietz Syndrome, Wiskott-Aldrich syndrome, autoimmune lymphoproliferative syndrome, Immunodysregulation, Polyendocrinopathy, Enteropathy, X-linked (IPEX) Syndrome, Comel-Netherton syndrome, and Severe Dermatitis, Multiple Allergies, and Metabolic Wasting (SAM) Syndrome (45). These IEIs underscore the importance of eosinophilia not just as a biomarker of allergic inflammation, but as a clue to underlying PID, particularly when combined with other signs of immune dysfunction such as recurrent infections, high IgE, and autoimmunity (46). A study by Lee et al. identified 140 variants in 59 genes in patients with IHES. The most frequently mutated genes were NOTCH1 (26.7%), SCRIB, and STAG2 (16.7%), and SH2B3 (13.3%). Network analysis highlighted 21 candidate genes, including BIRC3, BRD4, CSF3R, DNMT3A, EGR2, EZH2, FAT4, FLT3, GATA2, IKZF, JAK2, MAPK1, MPL, NF1, PTEN, RB1, RUNX1, TET2, TP53, and WT1, with MAPK1, RUNX1, GATA2, NOTCH1, and TP53 being major genes due to their high number of linkages to the eosinophilopoietic pathway (47). Höglund et al. performed whole-exome sequencing and identified 220 genes associated with eosinophil count. Seven genes driven by rare variants were ALOX15, CSF2RB, IL17RA, IL33, JAK2, S1PR4, and SH2B3. Two novel genes, NPAT and RMI1, were also identified as new eosinophil loci (48).

#### 2.2.6 Eosinophilic asthma flow

**Figure 6:**
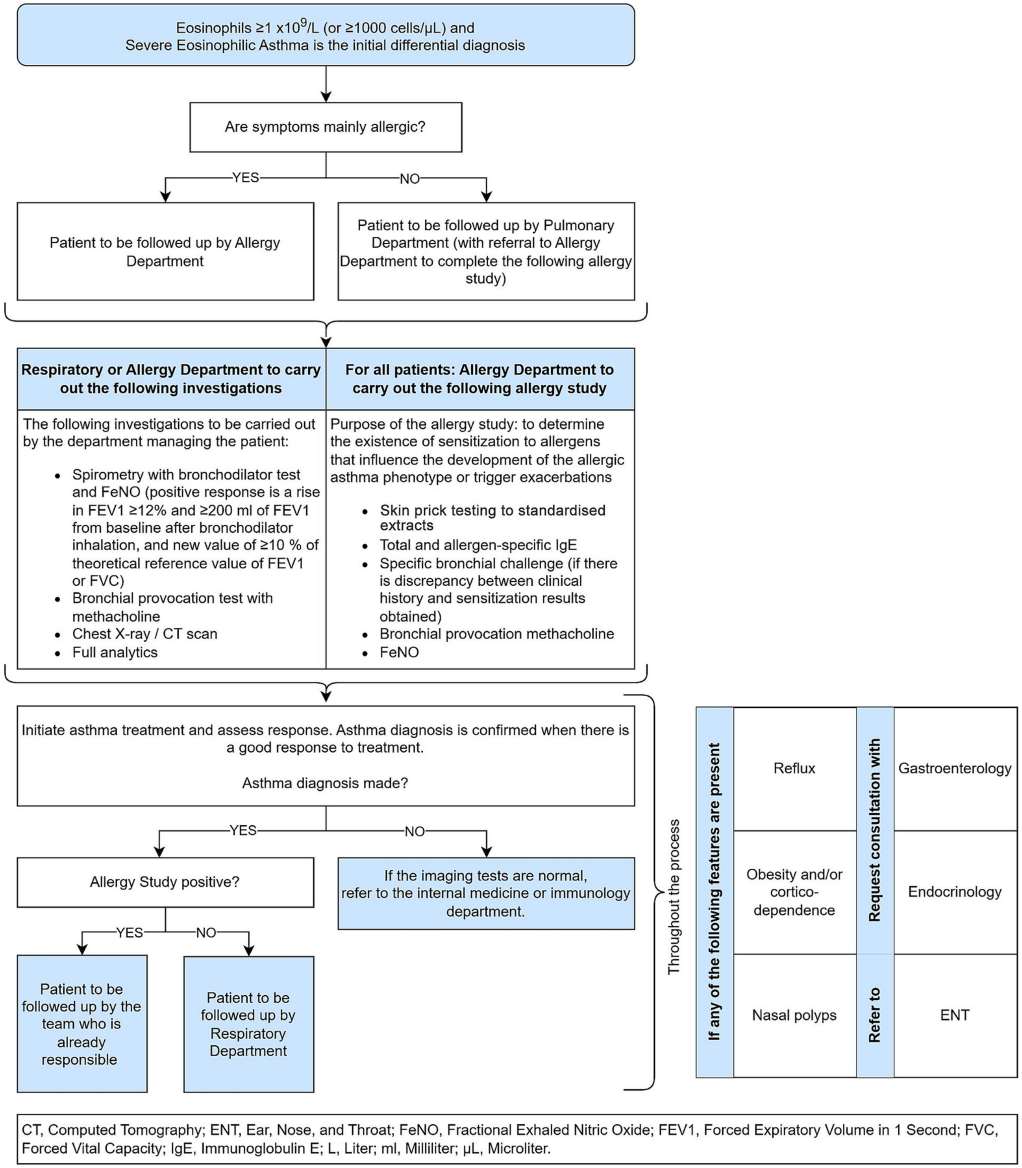
Eosinophilic asthma flow, from initial suspicion to confirmed diagnosis.

In a patient with suspected asthma and relevant eosinophilia the process outlined in Figure 6 shall be established, taking the patient from a suspicion of eosinophilic asthma to confirmation of the diagnosis.

After completing the patient flow as outlined in Figure 6, a patient with confirmed severe eosinophilic asthma will be jointly reviewed by members of the multidisciplinary severe asthma unit (MSAU). The MSAU will comprise physicians from Respiratory Medicine, Allergy Medicine, Ear nose and Throat Medicine (ENT), Clinical Immunology and Pediatrics, as well as hospital pharmacy.

Follow-up visits with a member of the MSAU (allergist or pulmonologist) will be scheduled every 6 months to monitor lung function, adherence to treatment and inhalation technique and, if necessary due to lack of response to treatment, to review the diagnosis.

In contrast, if Eosinophilic asthma is not confirmed by the unit, the presence of other respiratory pathologies will be reviewed again.

#### 2.2.7 Chronic rhinosinusitis with nasal polyposis flow

Although CRSwNP primarily falls under ENT expertise, patients often have associated with other lower respiratory tract conditions, most commonly asthma (49). This duality necessitates establishing a clear diagnostic pathway for optimal patient management focusing on nasal polyps.

**Figure 7:**
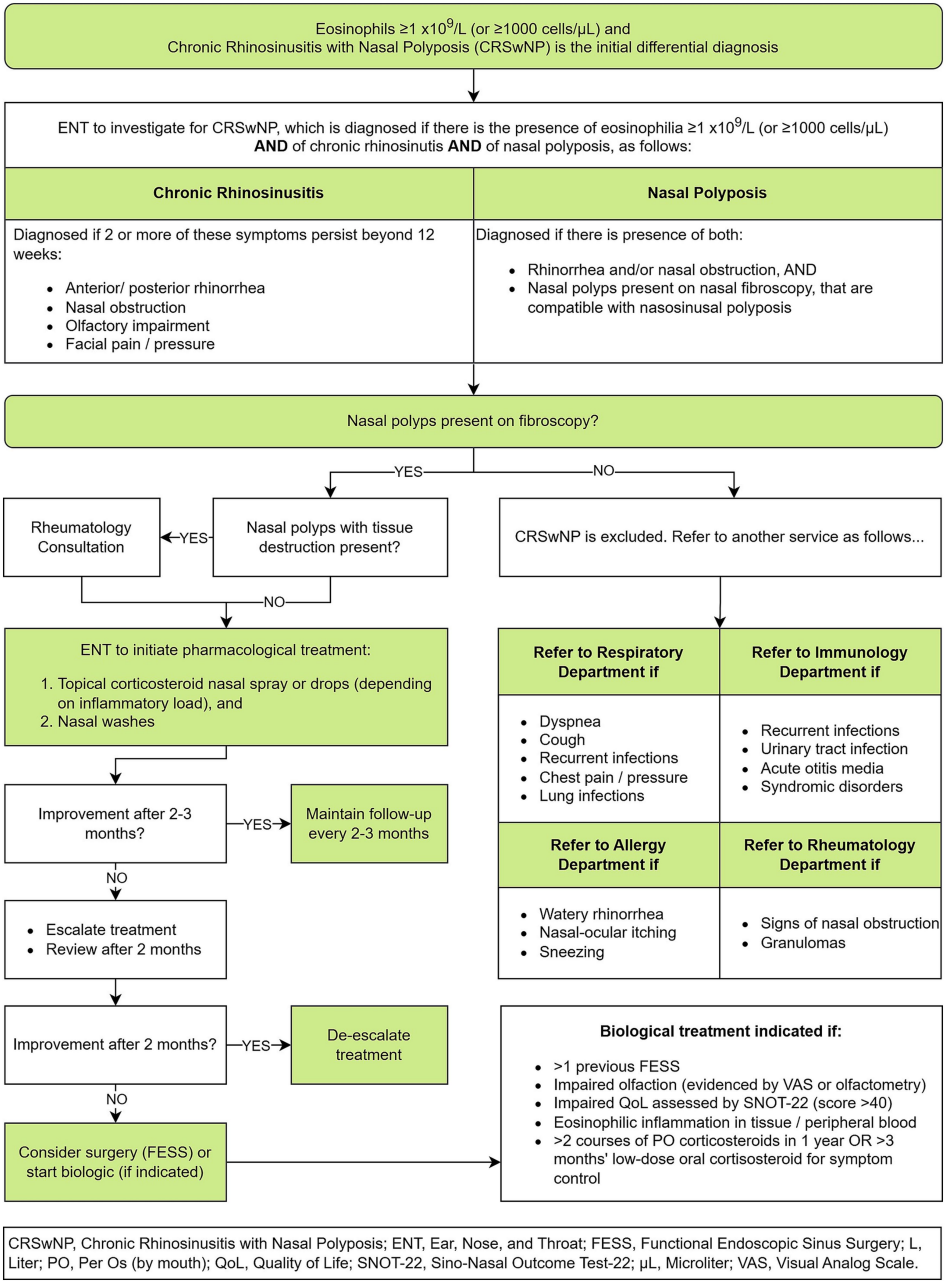
Chronic rhinosinusitis with nasal polyposis flow, from initial suspicion to confirmed diagnosis and management.

In a patient with suspected CRSwNP the process outlined in Figure 7 shall be established, taking the patient from a suspicion of CRSwNP to confirmation of the diagnosis and its management.

#### 2.2.8 Dual diagnoses flows

The management of patients with a dual diagnosis of two eosinophilic pathologies shall now be outlined.

For patients with CRSwNP and EGPA, follow-up will take place as per the EGPA flow (Figure 4). In case of uncontrolled rhinosinusitis symptomatology, a consultation with ENT will take place.

Patients with SEA and CRSwNP should be reviewed by the MSAU unit every 4–6 months, unless the patient has visited the ED, in which case they will be reviewed earlier. Before the MSAU meets, appropriate investigations to assess the patient’s evolution will have been requested by the physician in charge of the case and completed. If both pathologies are well controlled, the patient will be followed up again every 4–6 months. In contrast, if either of both pathologies are poorly controlled, the patient will be re-evaluated in a multidisciplinary manner and consideration will be given as to whether treatment modification is necessary or, in the case of CRSwPN, a surgical intervention.

## 3 Actionable recommendations

We recommend that this patient flow be piloted in other hospitals, with the aims to render financial resources more efficient, to decrease time from initial presentation to final diagnosis, and ultimately, to improve morbidity and mortality rates.

Outcomes should be evaluated by measuring the following parameters before and after implementation of the new flow process:

- total number of patients who receive a formal diagnosis of SEA/CRwNP/EGPA/HES/PID, and
- time from first hospital presentation until formal diagnosis.

## 4 Discussion

The introduction of a patient flow pathway for managing persistent eosinophilia and hypereosinophilia of unknown cause represents a substantial advancement in the clinical approach to these underdiagnosed and important pathologies. We aimed to streamline the process from patient presentation to diagnosis and initial management, with a specific focus on early identification and expedited referral circuits. We have used triage stratification consistent with the risk-adapted therapy approach recommended by the WHO, which aims to mitigate eosinophil-mediated organ damage while tailoring the urgency of intervention based on the severity and presence of organ involvement (23, 24).

Our findings indicate that the implementation of a structured patient flow pathway can significantly enhance the timely diagnosis and management of patients presenting with eosinophilia of unknown cause. The integration of multidisciplinary collaboration ensured a comprehensive evaluation and management approach. This multidisciplinary strategy is critical given the complex and often ambiguous presentations of eosinophilic disorders.

Traditional approaches to the evaluation of eosinophilia are often fragmented, leading to delayed diagnoses, repeated emergency room visits, and inefficient use of resources due to uncoordinated, sequential referrals between specialties. In contrast, our proposed multidisciplinary circuit ensures early identification of severe cases and provides a consensus-based, protocolized flow that replaces this fragmented pathway with an integrated diagnostic approach.

Evidence from the literature supports the clinical and economic burden associated with diagnostic delays in EGPA, PID, and HES (7, 12, 42). By enabling parallel rather than serial specialist assessments and proposing a predefined referral structure, our model accelerates the diagnostic timeline. For example, Bell et al. estimated that delayed diagnosis in EGPA increases total healthcare costs significantly and contributes to avoidable morbidity (7). Likewise, PID diagnosis is often delayed by years, with up to 90% of patients undiagnosed in standard care settings (42); our early screening protocol aims to capture these patients at the point of persistent eosinophilia detection, thus offering a marked advantage in timely treatment initiation.

Although formal outcome metrics from the current implementation are being collected, preliminary observations at our institution suggest a significant reduction in time to diagnosis (unpublished internal data) and improved patient satisfaction due to reduced diagnostic ambiguity. This contrasts with conventional approaches where diagnosis can take months or years. Future studies are planned to quantify these improvements using metrics such as diagnostic delay, rate of confirmed diagnoses, and healthcare utilization pre- and post-implementation.

Other of the key strengths of our proposed flow is its replicability in other hospital settings. The standardized pathway facilitates consistency in patient care, which is crucial for conditions with complex and often ambiguous presentations. By reducing variability in diagnostic and management practices, our model aims to decrease preventable morbidity and mortality associated with delayed or inappropriate treatment.

In line with previous research, our study supports the importance of a multidisciplinary approach in managing eosinophilic disorders. Thomsen et al. emphasized the need for coordinated care among multiple specialties for hypereosinophilic syndromes, which aligns with our findings and underscores the value of collaborative efforts in patient management (50). However, our study advances the field by offering a structured and replicable pathway that can be standardized across various clinical settings.

Eosinophilia can result by a wide range of conditions across various specialties (e.g., rheumatology, allergy, dermatology, gastroenterology, pulmonary medicine, hematology, infectious disease, and immunology). A single physician may lack the comprehensive expertise required to diagnose and manage complex or multi-systemic cases effectively leading to delays in diagnosis and treatment, with the potential for irreversible organ damage (50).

Unlike standard medical textbooks or clinical platforms such as UpToDate, which typically address eosinophilia through a sequential physician-led process, our proposed patient flow introduces a transversal, multidisciplinary model from the outset. Conventional management often begins with an isolated assessment, usually in primary care or the emergency department—followed by successive referrals to various specialists, which can be time-consuming and fragmented. In contrast, our flowchart establishes clear clinical criteria and predefined referral routes, allowing early activation of coordinated diagnostic processes across multiple departments. It also integrates practical screening tools and context-adapted algorithms designed for real-world hospital settings, facilitating early identification of severe or underdiagnosed conditions such as PID or eosinophilic vasculitis.

This proactive, protocol-driven approach not only accelerates diagnosis but also avoids/reduces tests duplication, minimizes waiting times, and enhances healthcare system efficiency and sustainability. Additionally, it supports the use of targeted therapies and personalized treatment plans, ultimately improving patient outcomes (51). However, coordinating a multidisciplinary team is logistically challenging and resource-intensive, requiring effective communication and collaboration among specialists—factors that may not always be feasible in all healthcare settings.

The economic implications of our model are noteworthy. Streamlining and coordinating the patient flow process can potentially reduce unnecessary healthcare costs. Repeated diagnostic tests, iterative visits, and nonspecific treatments not only increase patient suffering but also impose a significant financial burden on healthcare systems. Our pathway’s emphasis on early identification and management can mitigate these costs, contributing to more efficient use of healthcare resources.

Despite the promising results, this study has limitations. As a pilot study, further validation in diverse healthcare environments is necessary to confirm the effectiveness of the pathway. Follow-up studies should assess the sustained impact of the patient flow pathway on patient outcomes and healthcare costs. Additionally, larger studies are needed to confirm these preliminary findings.

Future research should focus on broader application and validation of this pathway in various clinical settings. Investigating the long-term impact on patient outcomes and healthcare costs, as well as patient and provider satisfaction, can provide insights for further refinement and optimization of the pathway.

In conclusion, the development and implementation of a structured patient flow pathway for eosinophilia of unknown cause represents a significant step forward in improving patient outcomes and optimizing healthcare resource utilization. This model serves as a template for other healthcare institutions seeking to enhance the management of eosinophilic diseases. Continued research and adaptation of this pathway will be essential to fully realize its potential benefits and ensure its applicability across diverse hospitals and populations.

## Data Availability

The original contributions presented in the study are included in the article/supplementary material, further inquiries can be directed to the corresponding author.
